# Biosurfactant-Based Interventions for Improving Lung Function and Controlling Inflammation

**DOI:** 10.1007/s11095-026-04202-4

**Published:** 2026-09-18

**Authors:** Nathalia Marton Sant’Ana, Paula Rezende-Teixeira

**Affiliations:** Post-Graduation Program in Health Sciences, Santo Amaro University, Professor Eneas de Siqueira Neto Street, 340. Jardim das Imbuias, São Paulo, SP 04829-300 Brazil

**Keywords:** antimicrobial activity, antiviral activity, biosurfactant, pulmonary inflammation, respiratory diseases

## Abstract

Biosurfactants are promising biotechnological compounds for respiratory diseases due to their ability to act as antimicrobials, antivirals, and anti-inflammatories, as well as their capacity to reduce pulmonary surface tension. *In vitro* evidence shows that molecules such as surfactin, rhamnolipids, sophorolipids, and MELs can partially mimic natural pulmonary surfactant, promote alveolar stabilization, and improve respiratory mechanics. Furthermore, these compounds modulate essential inflammatory pathways, including NF-κB, MAPK, and inflammasomes (NLRP3 and AIM2), leading to decreased production of the pro-inflammatory cytokines IL-1β, TNF-α, and IL-6. Activity against *Pseudomonas aeruginosa*, *Staphylococcus aureus*, *Mycobacterium tuberculosis*, respiratory fungi, and enveloped viruses support their use as therapeutic agents in infectious or inflammatory conditions of the respiratory tract. Despite their potential, several challenges persist, particularly the high production costs, difficulties in structural standardization, the limited stability of inhalable forms, and the lack of clinical trials evaluating safety and efficacy in humans. The development of more advanced disease prediction models, such as pulmonary organoids and "lung-on-a-chip" systems, emerges as an essential path for translational evaluation. Thus, although many obstacles remain, this body of evidence demonstrates that biosurfactants represent an innovative and promising platform for improving lung function or modulating inflammatory processes in various respiratory diseases.

## Introduction

Respiratory diseases are one of the leading causes of morbidity and mortality globally, representing one of the greatest public health challenges of the twenty-first century, including Chronic Obstructive Pulmonary Disease (COPD), asthma, pulmonary fibrosis, and respiratory infections [1]. COPD is currently the fourth leading cause of death worldwide, responsible for approximately 3.5 million deaths in 2021, according to the World Health Organization [2]. It affects more than 390 million people globally, with increasing prevalence due to population aging and continued exposure to risk factors such as smoking and air pollution [3]. While asthma is one of the most prevalent chronic diseases in the world, affecting approximately 262 million individuals and causing about 455,000 deaths annually [4]. Although asthma is treatable, inequalities in access to diagnosis and medication contribute to high hospitalization and mortality rates in low-income countries [5, 6]. Pulmonary fibrosis, especially Idiopathic Pulmonary Fibrosis (IPF), has a lower incidence but a significant clinical impact. Its prevalence is estimated to range between 13 and 20 cases per 100,000 people, with high mortality: the average survival after diagnosis is 3 to 5 years [7]. Finally, respiratory infections, including influenza, pneumonia, and emerging viral diseases, remain among the leading causes of preventable death. Pneumonia is responsible for approximately 2.5 million deaths annually, including 740,000 children under 5 years old [6, 8]. Despite presenting distinct etiologies and pathophysiological mechanisms, these conditions share processes relevant to pulmonary homeostasis, including inflammation, altered epithelial barrier function, impaired respiratory mechanics, and, in certain conditions, the persistence of infectious agents.

The therapies currently used to treat respiratory diseases, such as anti-inflammatory drugs, bronchodilators, corticosteroids, antibiotics, and antifibrotics, represent important therapeutic advances; however, they have limitations that restrict and compromise their long-term effectiveness, such as adverse effects, increasing antimicrobial resistance, and heterogeneous therapeutic response, particularly in patients with severe disease, a refractory phenotype, or persistent infections, reinforcing the need for novel strategies [9–20]. Thus, the need to develop new therapeutic and preventive approaches, safer, more effective, and more accessible capable of improving lung function, combating resistant infections, and reducing chronic inflammation, is imminent.

**Fig. 1:**
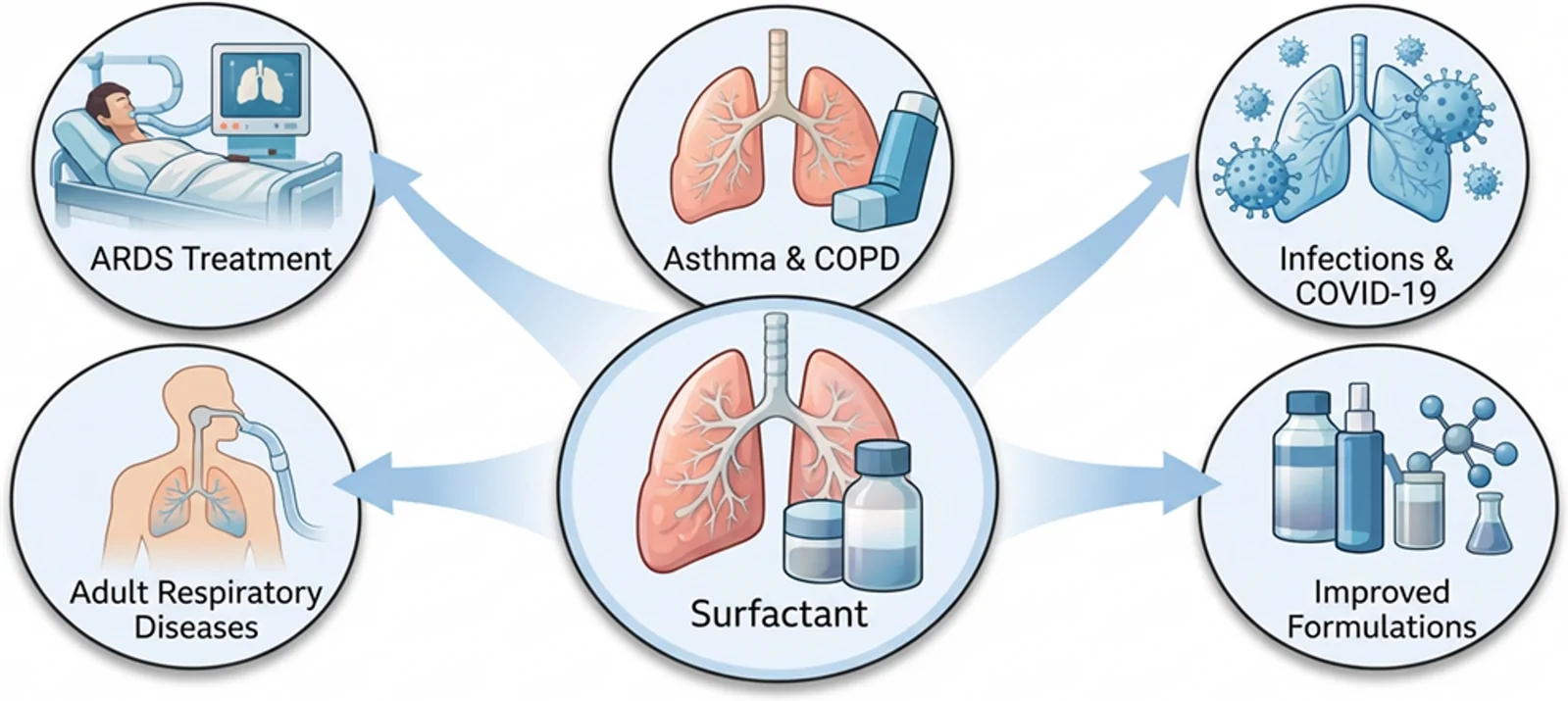
Emerging clinical applications for next-generation pulmonary surfactants. Overview of key therapeutic areas where novel pulmonary surfactants may provide clinical benefits, including ARDS in adults, asthma and COPD, severe viral infections (e.g., COVID-19), new enhanced or combined formulations, and nanotechnology.

In this context, biosurfactants emerge as molecules of biotechnological interest due to a combination of physicochemical and biological properties potentially relevant for respiratory applications. Notable among them are rhamnolipids, surfactin, sophorolipids, and mannosylerythritol lipids (MELs), which exhibit surfactant, antimicrobial, and anti-inflammatory activities described in various experiments models. Furthermore, their biodegradability and potentially favorable toxicity profiles, depending on structure, concentration, and purity, underscore the interest in these molecules for the development of new therapeutic approaches [21, 22]. Preclinical evidence indicates that biosurfactants can exert multiple effects relevant to respiratory diseases, including the modulation of pulmonary surface tension, antimicrobial and antibiofilm activity, and the regulation of inflammatory responses [23, 24]. These properties may contribute to the preservation of alveolar stability, the improvement of respiratory mechanisms, the control of respiratory pathogens, and the attenuation of excessive inflammation [21, 25–27]. Furthermore, their biodegradability and generally favorable safety profiles support their potential for development as inhalable therapeutic agents, although challenges regarding production, structural standardization, formulation stability, toxicity, and clinical validation persist (Fig. 1).

Considering this evidence, this review seeks to critically synthesize the available scientific data on biosurfactants as potential therapeutic agents for improving lung function and modulating inflammatory processes in the respiratory diseases. The review pays special attention to effects on pulmonary physiology, immunomodulatory and anti-inflammatory mechanisms, antimicrobial and antiviral activities, as well as potential applications in respiratory therapy. By integrating evidence from *in vitro* studies and preclinical models, the work identifies gaps in current knowledge and discusses challenges and prospects for the clinical and biotechnological development of biosurfactant-based interventions.

## Biosurfactants: Characteristics, Classification, and Functional Properties

Biosurfactants are amphiphilic molecules produced by microorganisms, capable of reducing surface tension in different biological systems, which supports their growing interest in biomedical applications. Structurally, they can be composed of lipids, carbohydrates, proteins, or combinations thereof, giving them diverse physicochemical and biological properties. Among the main classes studied with therapeutic potential are rhamnolipids, surfactin, MELs, and sophorolipids [28–30].

### Rhamnolipids

Rhamnolipids are produced primarily by *Pseudomonas aeruginosa* and are glycolipids composed of one or two rhamnose units linked to fatty acid chains. These compounds exhibit a high capacity for reducing surface tension, strong antimicrobial activity, especially against Gram-negative bacteria, and notable action in disrupting biofilms, in addition to immunomodulatory properties and low toxicity at controlled concentrations [31–35].

### Surfactin

Surfactin, a cyclic lipopeptide produced by *Bacillus subtilis*, is considered one of the most potent natural surfactants, capable of reducing the surface tension of water to values close to 27 mN/m [36, 37]. Its amphiphilic structure confers intense antimicrobial, antiviral, and anti-inflammatory activity [38–40]. Studies demonstrate that surfactin can modulate pathways such as NF-κB and MAPK and has potential for pulmonary applications due to its ability to partially mimic natural alveolar surfactant [40].

### Mannosylserythritol Lipids (MELs)

Mannosylserythritol lipids (MELs) are glycolipid-type biosurfactants produced predominantly by yeasts of the genus *Pseudozyma* (currently reclassified as *Moesziomyces*), including *Pseudozyma antarctica*. These compounds exhibit high biodegradability, low cytotoxicity, and excellent surfactant activity, characteristics that make them particularly attractive for biomedical and pharmaceutical applications [41, 42]. *In vitro* studies demonstrate that MELs display high biocompatibility, as well as anti-inflammatory properties and potential for favorable interaction with biological membranes, reinforcing their safety for therapeutic applications [43, 44]. MELs exhibit strong wetting capacity and high affinity for biological surfaces, as well as anti-inflammatory and antioxidant properties, characteristics that make them very promising for pharmaceutical applications, including sensitive respiratory formulations, where biocompatibility, stability, and low cytotoxicity are essential requirements [41–43].

### Sophorolipids

Sophorolipids are glycolipid-type biosurfactants produced mostly by yeasts such as *Starmerella bombicola* [45]. These compounds occur in lactonic and acidic forms, which exhibit distinct physicochemical and biological properties, influencing their biological activity and pharmaceutical applicability [45, 46]. Sophorolipids exhibit excellent antimicrobial and antibiofilm activity, acting against Gram-positive and Gram-negative bacteria, as well as clinically relevant fungi [47, 48]. Additionally, studies demonstrate its immunomodulatory effects, including the modulation of inflammatory cytokine production, together with high cellular compatibility and low cytotoxicity in *in vitro* models [48–50]. This structural versatility, combined with biodegradability and biocompatibility, favors its application in controlled release systems and medical formulations, including potential applications in the respiratory tract [51, 52].

### Functional Properties

From a functional perspective, these biosurfactants exhibit biological properties of great relevance for pulmonary applications. Firstly, they show antimicrobial activity against respiratory pathogens, including Gram-positive and Gram-negative bacteria and fungi, in addition to acting in the disruption of biofilms, one of the greatest therapeutic challenges in chronic pulmonary diseases [33, 43, 48, 53]. Literature shows that biosurfactants such as rhamnolipids, surfactin, and sophorolipids exhibit strong antimicrobial activity against relevant respiratory pathogens, including *Pseudomonas aeruginosa* [54], *Staphylococcus aureus* [54, 55], *Mycobacterium tuberculosis* [56] and fungi such as *Aspergillus niger* [54, 57]. Studies show that these compounds disrupt microbial membranes, inhibit biofilm formation, and enhance the action of antibiotics [48, 55]. Additionally, the study by Wang *et al.* [48] is highly relevant as it systematically demonstrates that sophorolipids exhibit significant inhibitory activity against bacteria and fungi, including pathogens of clinical interest, reinforcing their potential as antimicrobial agents. By evaluating different structural variants, the work establishes structure–activity relationships that help to understand the molecular determinants of the biological efficacy of these compounds. The results presented, combined with the low cytotoxicity observed, support the applicability of sophorolipids as alternatives or therapeutic adjuvants in addressing antimicrobial resistance, with particular relevance for the development of biomedical and respiratory formulations [48].

Secondly, biosurfactants have the ability to reduce surface tension, an essential characteristic for alveolar stability, partially mimicking the behavior of natural pulmonary surfactant produced by type II pneumocytes [58]. Among these compounds, surfactin is particularly relevant as one of the most potent natural biosurfactants, capable of reducing the surface tension of water to values close to 27 mN/m, contributing to alveolar stabilization and the prevention of lung collapse in experimental models [36, 37]. To a reduced extent, MELs and sophorolipids also demonstrate relevant surfactant properties, with potential for biomedical and pulmonary applications, although with less surface tension reduction capacity when compared to surfactin [42, 59, 60]. Moreover, experimental evidence indicates that surfactin can modulate pulmonary inflammatory responses, reinforcing its therapeutic potential in the respiratory context [40].

Furthermore, many biosurfactants exhibit immunomodulatory and anti-inflammatory action, acting on the regulation of central signaling pathways of the inflammatory response, such as NF-κB, with a consequent reduction in the production of pro-inflammatory cytokines, including IL-1β, IL-6, and TNF-α. Experimental evidence indicates that this inflammatory modulation may be particularly beneficial in conditions characterized by chronic inflammation of the respiratory tract [40, 61]. Other studies demonstrate that different classes of biosurfactants can modulate critical inflammatory pathways, including NF-κB, MAPK, and Toll-like receptors (TLR2/TLR4), as well as positively interfering with inflammasome activation, especially NLRP3. In cellular and animal models, this modulation results in a significant reduction in the production of IL-1β, TNF-α, and IL-6, associated with decreased neutrophil infiltration and oxidative stress in lung tissues, reinforcing the therapeutic potential of these compounds in controlling respiratory inflammation [62, 63].

Finally, the low toxicity and high biodegradability of biosurfactants underscore their potential as safe and environmentally sustainable alternatives to synthetic surfactants [59, 60, 64]. Biosurfactants such as MELs and sophorolipids exhibit excellent biocompatibility profiles, low cytotoxicity, and rapid environmental biodegradation. Even rhamnolipids and surfactin, which exhibit higher biological activity, show controlled toxicity at therapeutic concentrations and are less persistent in the environment than synthetic surfactants. These findings reinforce their potential safety for respiratory formulations and medical applications [59, 60]. Thus, the set of physical and biological characteristics of these compounds supports their growing interest as therapeutic agents and adjuvants for interventions aimed at improving pulmonary function and controlling respiratory inflammation. Biosurfactants can increase the bioavailability and solubility of poorly water-soluble drugs, improving their administration and efficacy [65] (Table 1).

**Table 1:** Comparative overview of major biosurfactant classes and their physicochemical properties, biological activities, mechanisms of action, and potential pulmonary applications

Biosurfactant Type	Source (Microorganism/Origin)	Main Physicochemical Properties	Main Biological Activities	Mechanism of Action	Pulmonary Applications	Other Relevant Aspects
**Surfactin** (Cyclic Lipopeptides)	*Bacillus subtilis and other Bacillus species*	One of the most potent natural biosurfactants; reduction of surface tension of water from 72 to 27 mN/m (at 10–20 μM); amphiphilic structure	Antibacterial, antifungal, antiviral, antitumor, anti-inflammatory, and immunomodulatory activities	Interaction with cell membranes; pore formation; modulation of NF-κB/MAPK/TLR4 and inflammasome pathways; inhibition of viral membrane fusion	Potential for controlling respiratory infections and biofilms, reducing pulmonary inflammation	High biological activity at low concentrations; strong translational potential
**Sophorolipids** (Glycolipids)	*Starmerella bombicola*, *Candida apicola* and other yeasts	Good surfactant activity; high biodegradability; stability; emulsifying capacity	Antimicrobial, antifungal, antibiofilm, anti-inflammatory, and antioxidant	Interaction with cell membranes; alteration of membrane permeability; interference with microbial adhesion and biofilm formation	Potential for treating pulmonary infections and reducing biofilms; possible use in drug-delivery systems	Relatively well-established industrial production; properties depend on lactonic or acidic forms
**Rhamnolipids** (Glycolipids)	*Pseudomonas aeruginosa* and *Burkholderia* spp.	Excellent emulsifying capacity; high surface tension-reducing capacity; high interfacial activity; micelle formation	Antimicrobial, antibacterial, anti-biofilm, and immunomodulatory activities	Disruption of microbial membranes; interference with adhesion and biofilm extracellular matrix; modulation of inflammatory pathways	Potential against respiratory biofilms and multidrug-resistant bacterial infections; possible application in inhalable formulations	Production by *P. aeruginosa* requires rigorous purification strategies for pharmaceutical applications
**Mannosylerythritol Lipids (MELs)**	*Pseudozyma* spp.* (P. antarctica*, *P. aphidis)/ Moesziomyces* spp.	Excellent surfactant activity; relatively low cytotoxicity; high biodegradability and favorable interaction with cellular membranes	Antimicrobial, antifungal, antibiofilm, and immunomodulatory	Alteration of membrane organization and interaction with lipid components; interference with microbial adhesion	Potential for respiratory infections, biofilm control, and delivery systems for bioactive molecules	Low toxicity, high biodegradability, and a favorable safety profile, attractive for pharmaceutical applications

## Biosurfactants and Pulmonary Physiology

### Surface Tension Modulation and Alveolar Stability

Biosurfactants have attracted increasing interest in the pulmonary context due to their ability to mimic key properties of natural surfactant, playing relevant roles in respiratory mechanics and inflammatory control. Endogenous pulmonary surfactant, synthesized by type II pneumocytes, is a complex mixture mainly composed of phospholipids, predominantly dipalmitoylphosphatidylcholine (DPPC), and specific proteins known as surfactant proteins (SP-A, SP-B, SP-C, SP-D), whose central physiological function is to reduce alveolar surface tension, stabilize micro-alveoli, and facilitate gas exchange [66, 67]. Furthermore, surfactant proteins have an immunomodulatory role, participating in the regulation of the inflammatory and immune response in the lungs [68]. While SP-B and SP-C are responsible for the stability of the surfactant film and facilitate its rapid dispersion on the alveolar surface during respiratory cycles, in some respiratory pathologies, such as asthma, where the immune response is unbalanced, the function of these proteins may be altered, potentially exacerbating allergic inflammation and hyper-reactivity [58, 69]. Microbial biosurfactants, including surfactin, rhamnolipids, sophorolipids, and MELs, share an amphiphilic nature that gives them the ability to interact efficiently with air–liquid interfaces, exhibiting functional behavior like, though not identical to, that of pulmonary alveolar surfactant. This property may represent great potential for biomedical applications, particularly in the context of modulating surface tension and alveolar stability [38, 70, 71].

From a functional standpoint, the presence of surfactants can promote alveolar stabilization by reducing the surface elastic modules and facilitating alveolar reinflation during episodes of collapse [70, 72, 73]. However, mimicry is partial and faces significant translational limitations when it comes to biosurfactants. Interactions with surfactant proteins (SP-B/C), crucial for the compression/expansion dynamics of the alveolar film, are not yet fully characterized for biosurfactants; the stability of the film formed *in vivo*, susceptibility to inactivation by serum/plasma, and the possibility of cytotoxic or hemolytic effects (dependent on concentration and purity) require rigorous evaluation [38, 59].

### Impacts on Respiratory Mechanics and Pulmonary Function

Beneficial effects were demonstrated in preclinical studies investigating parameters of respiratory mechanics, oxygenation, and inflammatory injury, and consequently, these effects may translate into favorable changes in respiratory mechanics, including reduced pulmonary resistance, increased compliance, and improved gas exchange efficiency [74, 75]. In summary, biosurfactants can reproduce essential aspects of natural pulmonary surfactants, especially the reduction of surface tension, and they add antimicrobial and immunomodulatory properties that make them particularly attractive for pulmonary interventions. However, their clinical translation depends on robust proof of compatibility with the native surfactant system, a safe toxicological profile, and formulations capable of maintaining stability and activity in the alveolar environment [31, 38, 59, 72]. These characteristic properties of biosurfactants give these compounds the ability to stabilize alveoli, reduce alveolar collapse, and improve respiratory mechanics, which are critical factors for patients with respiratory diseases characterized by surfactant deficiency or exacerbated inflammation [70, 73]. Experimental studies showed that surfactin has a marked surfactant action, significantly reducing the surface tension of model systems and promoting more efficient alveolar expansion [55]. Studies in experimental models of acute lung injury showed that the administration of biosurfactants is associated with a reduction in pulmonary resistance, improved dynamic compliance, and decreased atelectasis, indicating direct and beneficial effects on respiratory mechanics [76, 77]. Additionally, the anti-inflammatory action of these compounds may contribute to the attenuation of pulmonary edema, reduction of inflammatory cell infiltration, and preservation of alveolar architecture, factors that, together, positively impact overall lung function and the efficiency of gas exchange [78, 79].

### Formulation, Administration, and Translational Challenges

In terms of formulation, the development of efficient purification strategies, as well as appropriate transport systems and administration routes, such as nebulization or intratracheal instillation, becomes essential. These strategies must maintain the activity of the biosurfactants while minimizing potential local toxic effects [38, 64]. Hybrid lipid formulations that combine biosurfactants with phospholipids or peptides that mimic pulmonary surfactants have shown potential to improve alveolar film dynamics, favoring the reduction of surface tension and the stability of alveoli in experimental models [38, 76, 77]. Additionally, the development of nanoparticles functionalized or coated with biosurfactants has been explored as a strategy to increase the stability of the therapeutic load, promote controlled release, and confer intrinsic antimicrobial activity to the delivery system, thus enhancing its effectiveness in complex pulmonary environments [52, 53, 60].

The inhalation route also allows for synergistic approaches, in which the co-administration of biosurfactants with antibiotics can increase penetration into pulmonary biofilms and reduce the doses required to achieve a therapeutic effect, while the combination with anti-inflammatory agents can attenuate the tissue inflammatory response without compromising local antimicrobial defense [53, 60, 80]. In this context, the use of advanced experimental models, including lung-on-a-chip platforms and human lung explants, can provide a more precise assessment of the interaction of these formulations with the human respiratory epithelium, allowing for better prediction of efficacy, safety, and the possibility of clinical translation [81–83]. Finally, conducting controlled clinical trials remains essential to confirm the therapeutic efficacy, safety, and translational feasibility of these approaches before any large-scale clinical application.

However, optimization of the purity and standardization of biosurfactants is still necessary, since structurally heterogeneous mixtures can significantly affect biological activity, reproducibility, and toxicological profile [60, 64]. Furthermore, ensuring physicochemical stability during nebulization is essential, considering factors such as maintaining surface tension, aggregate formation, molecular degradation, and interaction with inhaler devices. Other factors are also important, such as validating whether the atomization energy and the ideal average aerodynamic size of the particles (MMAD between 1 and 5 µm) ensure efficient alveolar deposition without inducing bronchospasm or local irritation [84, 85]. Specific toxicological studies are equally indispensable to evaluate bronchial irritation, hemolysis, local immunogenicity, interference with endogenous pulmonary surfactant, and pulmonary pharmacokinetics, which represent critical aspects for the safety of inhalable formulations [60, 77]. Rigorous purification procedures to remove endotoxins and microbial residues are essential, especially considering the microbial origin of these compounds [38, 64].

Recommended experimental strategies include comparative *in vitro* assays using 2D and 3D respiratory epithelium models and biofilm models, as well as ex vivo studies with human lung explants and bronchoalveolar lavage fluids, in addition to *in vivo* models for integrated evaluation of lung deposition, antimicrobial efficiency, respiratory mechanics, and inflammatory response [82, 83]. The use of lung-on-a-chip platforms and stability assays in commercial nebulizers has been identified as a strategic tool to accelerate translational prediction and reduce failures in advanced clinical stages [81, 82]. In parallel, defining therapeutic windows, safe and effective doses, and demonstrating clinical advantages over synthetic surfactants or conventional therapies will be crucial for the progression of these approaches towards controlled clinical trials [60, 84].

Overall, biosurfactants in inhalable formulations offer a promising platform for multifunctional pulmonary treatment, but require rigorous pharmaceutical development, robust toxicological evaluation, and clear demonstration of clinical benefit to overcome regulatory and operational barriers.

## Immunomodulatory and Anti-inflammatory Effects of Biosurfactants

Biosurfactants exhibit a range of anti-inflammatory mechanisms that position them as promising therapeutic candidates for respiratory diseases. Several studies have demonstrated that compounds such as surfactin, rhamnolipids, and sophorolipids exert potent immunological modulation, acting on both the innate immune response and chronic inflammation pathways.

### Modulation of NF-κB and MAPK Signaling

**Fig. 2:**
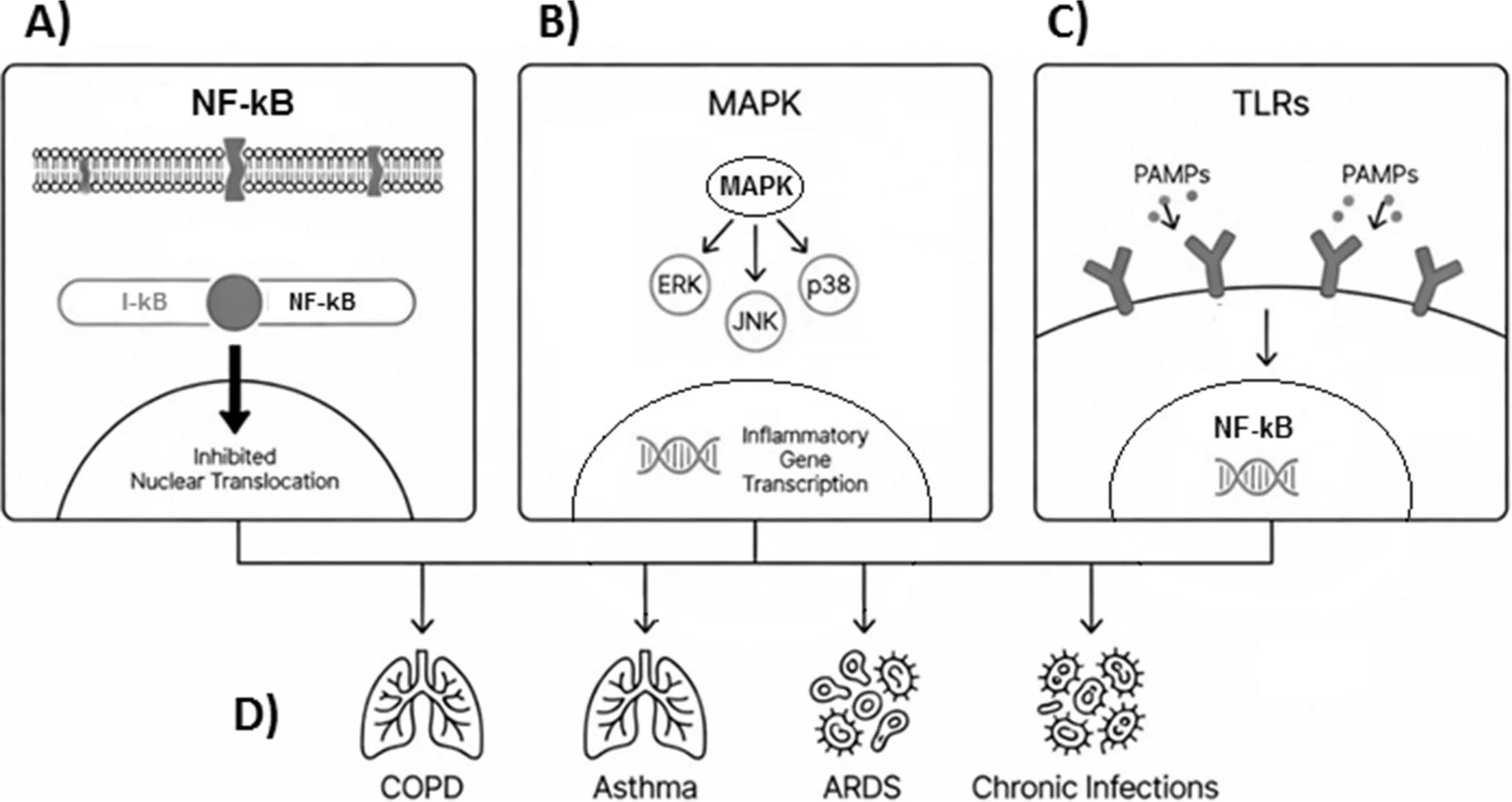
Schematic representation of the different cell signaling pathways (NF-κB, MAPK, and Toll-like receptors—TLRs) that can be modulated by biosurfactants in the pulmonary inflammatory response. (**A**) In the NF-κB pathway, the association with I-κB is maintained, inhibiting nuclear translocation and consequently reducing the transcription of pro-inflammatory genes. (**B**) In the MAPK pathway, the modulation of kinases such as ERK, JNK, and p38 interferes with the regulation of the transcription of inflammatory genes. (**C**) The interaction with TLRs and the recognition of PAMPs impact the activation of the innate immune response. (**D**) Inflammatory processes involved in respiratory diseases such as chronic obstructive pulmonary disease (COPD), asthma, acute respiratory distress syndrome (ARDS), and chronic infections.

Various preclinical evidence showed that biosurfactants, especially surfactin, rhamnolipids, sophorolipids, and MELs, apply a potent anti-inflammatory action by modulating central pathways of innate immunity, such as NF-κB, MAPK, and Toll-like receptors (TLRs). These mechanisms are fundamental in triggering and maintaining pulmonary inflammation, making them relevant therapeutic targets in diseases such as COPD, asthma, acute respiratory distress syndrome (ARDS), and chronic infections (Fig. 2).

Scientific evidence has shown that surfactin was able to reduce LPS-induced nitric oxide production in RAW264.7 cells and primary macrophages, an effect associated with the modulation of NF-κB pathway signaling [86]. Park and Kim (2009) also demonstrated that surfactin can modulate the immunostimulatory function of macrophages by blocking the NK-κB, MAPK, and Akt pathways. Using thioglycolate induced mouse peritoneal macrophages, the expression of surface molecule, cytokine production, phagocytosis, ability to induce T cell activation via mixed lymphocyte response and underlying signaling pathways were evaluated. Surfactin significantly suppressed lipopolysaccharide-induced CD40, CD54, CD80, and MHC-II expression, but not CD86 and MHC-I, indicating that surfactin exhibited potent immunosuppressive activity, with relevant therapeutic implications for the treatment of autoimmune diseases, allergic conditions, arthritis, diabetes, and in the context of transplantation [78].

Thus, in models of acute lung injury induced by LPS, endotoxins, or pathogens, we can observe the following evidence: 1) reduction of neutrophilic infiltration and pulmonary edema, where surfactin and rhamnolipids reduce the accumulation of neutrophils in bronchoalveolar lavage and decrease extravasated plasma proteins, indicating stabilization of the epithelial barrier and less tissue damage [62, 86]. 2) Reduction of pro-inflammatory cytokines (TNF-α, IL-1β, IL-6), observed through the negative modulation of NF-κB, MAPKs, and TLR4, which limits the inflammatory cascade, rapidly reducing the pro-inflammatory profile typical of acute inflammation [63].

In inflammatory models characterized by excessive NF-κB activation, particularly in LPS-stimulated macrophages, surfactin attenuates NF-κB signaling and reduces the expression of inflammatory mediators downstream in the signaling cascade. Conversely, in immature dendritic cells, surfactin can activate NF-κB signaling, contributing to cellular maturation and immune activation. Xu *et al.* [87] showed that the level of IκB-α in surfactin treated DCs was significantly reduced, while the nuclear level of p65 increased, indicating that the nuclear factor kappa B (NF-κB) signaling pathway may play an important role in surfactin induced DC maturation. In this context, surfactin can induce the transformation of immature DCs into mature cells, conferring greater capacity for migration and proliferation, and in this process, the NF-κB signaling pathway would be involved. This would suggest a possible underlying mechanism for the immunoprotective effects induced by surfactin [87]. It is important to highlight that the effects of surfactin on NF-κB signaling appear to be context-dependent rather than uniformly inhibitory. In LPS-stimulated macrophages, surfactin attenuates NF-κB activation and reduces downstream inflammatory mediators; conversely, in immature dendritic cells, surfactin can promote NF-κB activation, as indicated by reduced IκB-α levels and increased nuclear p65, thereby contributing to the maturation of these cells. These seemingly divergent effects may reflect differences in cell type, basal activation state, inflammatory stimulus, exposure conditions, and the biological response required by each cellular context.

Biosurfactants also appear to modulate the MAPK pathway, interfering with the phosphorylation of ERK1/2, JNK, and p38, proteins essential for amplifying the inflammatory response. Surfactin, for example, reduced the activation of these kinases in models of acute lung injury, which is directly linked to lower expression of IL-6 and chemokines responsible for neutrophil recruitment [88].

### Toll-like Receptors and Innate Immunity Signaling

The anti-inflammatory activity of surfactin in lipopolysaccharide (LPS)-activated macrophages showed reduced expression of the TLR4 protein, likely indicating that the surfactin-mediated signaling pathway is involved with TLR4 [40]. Among other immunological activities of surfactin are its ability to trigger defense responses related to the immune system and enhance humoral and cellular immune responses. Xu *et al.* [87] showed that surfactin appears to be able to induce dendritic cell (DC) maturation *in vitro*. After stimulation with 20 μg/ml of surfactin for 24 h, DCs acquired morphological and phenotypic characteristics of a mature state, exhibiting an increased shape index and elevated expression of major histocompatibility complex II (MHCII) and CD40. Furthermore, surfactin also induced DCs to release IL-6 and tumor necrosis factor alpha (TNF-α), indicating that the DCs were functionally mature.

Another modulated pathway involves TLRs, particularly TLR2 and TLR4, fundamental receptors in the response to respiratory pathogens and bacterial components. Biosurfactants can attenuate TLR4-mediated pro-inflammatory signaling, reducing responses to lipopolysaccharides and limiting the subsequent activation of NF-κB and MAPK [78]. This results in lower production of TNF-α, IL-1β, and IL-6, cytokines closely linked to alveolar damage and worsening respiratory mechanics. Taken together, these effects suggest that biosurfactants act not only as surface-active agents but also as multifunctional modulators of pulmonary inflammation, offering therapeutic potential in conditions where hyperactivation of these pathways plays a central role.

### Inflammasome Modulation

**Fig. 3:**
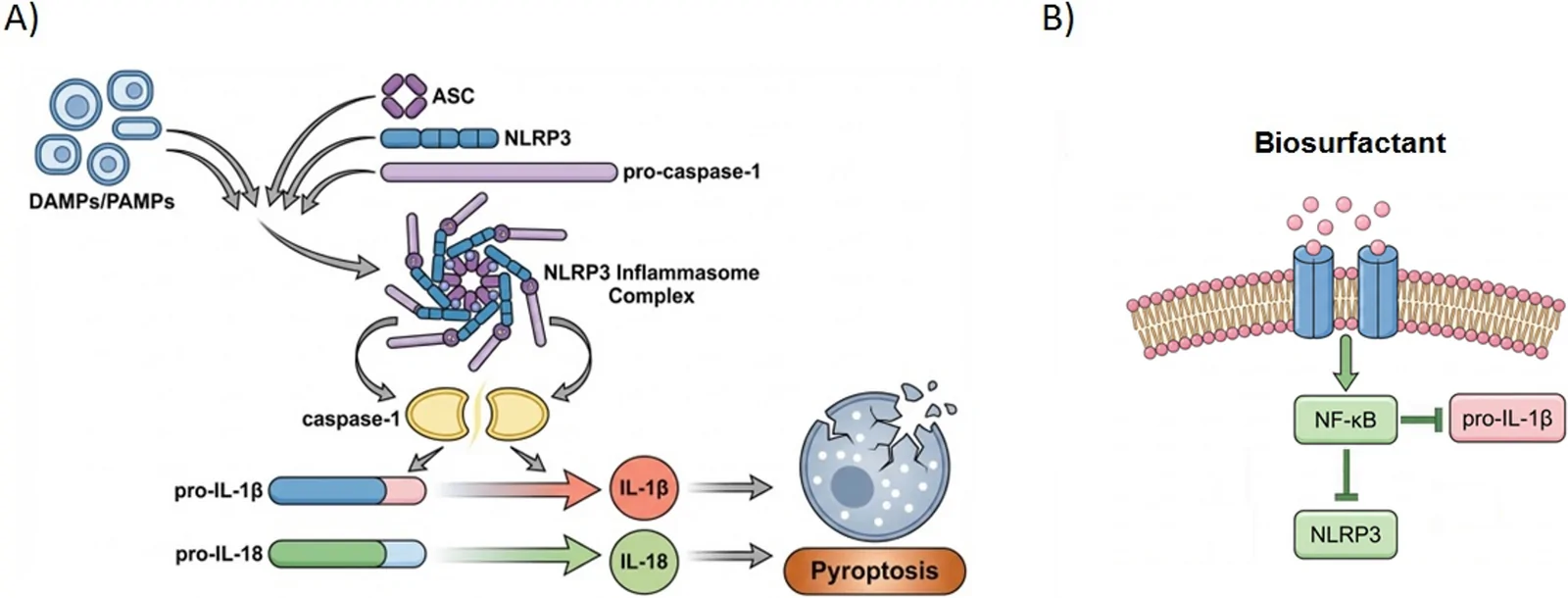
Biosurfactants modulating inflammasome activation and inflammatory signaling pathways. (**A**) Canonical activation of the NLRP3 inflammasome occurs through damage-associated molecular patterns (DAMPs) or pathogen-associated molecular patterns (PAMPs). These patterns recruit the adaptor protein ASC and pro-caspase-1, culminating in the activation of the NLRP3 inflammasome, which leads to the activation of caspase-1, which cleaves the inactive precursors pro-IL-1β and pro-IL-18 into their mature forms, IL-1β and IL-18. The release of these pro-inflammatory cytokines promotes inflammatory signaling and induces pyroptotic cell death. (**B**) Proposed anti-inflammatory mechanism for biosurfactants occurs when the biosurfactant interacts with the plasma membrane and inhibits NF-κB activation. This effect reduces the transcription of pro-IL-1β and NLRP3, and consequently the assembly of the inflammasome and the production of inflammatory cytokines.

Gan *et al.* [62], using *in vitro* and *in vivo* immunization studies, demonstrated that surfactin acted as an agonist, inducing the production of mitochondria dependent reactive oxygen species (ROS) to activate MAPK, NF-κB, and inflammasome pathways in macrophages. The study provided insights into mechanism of action of surfactin in triggering the immune response, primarily acting as an important adjuvant. Surfactin penetrated antigen presenting cells to induce ROS, increasing cytokine production and promoting the expression of co-stimulatory molecules. Evidence indicated that surfactin can suppress IL-1β maturation by inhibiting caspase-1 activation and NLRP3 inflammasome assembly in cell models, suggesting a direct role in containing inflammatory processes mediated by cytosolic sensors [62]. Inflammasomes (e.g., NLRP3, AIM2) represent central platforms in the detection of DAMPs/PAMPs and the activation of caspase-1, resulting in the maturation of IL-1β/IL-18 and pyroptosis, processes that amplify pulmonary inflammation in ARDS, COPD, infections, and chronic diseases [89, 90] (Fig. 3A).

Emerging evidence and data indicate that biosurfactants, particularly surfactin, rhamnolipids, and some glycolipids, can modulate inflammasomes through multiple complementary mechanisms. These include reducing the priming signal, where biosurfactants attenuate the activation of pattern recognition receptors (TLR4/TLR2) and block pathways such as NF-κB and MAPK in macrophages and epithelial cells, thus decreasing the transcriptional expression of pro-IL-1β and NLRP3 necessary for inflammasome priming [59, 62, 78] (Fig. 3B). The multifactorial profile of biosurfactants, blocking priming via TLR/NF-κB, reducing mitochondrial oxidative stress, antimicrobial/anti-biofilm action, and possible direct inhibition of inflammasome assembly, positions these compounds as promising candidates for modulating inflammasome responses (NLRP3/AIM2) in pulmonary diseases.

### Reduction of Oxidative Stress and Pro-inflammatory Cytokines

Scientific evidence has shown that the sophorolipid biosurfactant in LPS-induced RAW264.7 cells, where there was a reduction in the release of inflammatory cytokines, including interleukin-6 (IL-6) and tumor necrosis factor-α (TNF-α) [63]. Similarly, the anti-inflammatory activity of surfactin in lipopolysaccharide (LPS)-activated macrophages demonstrated to inhibit the increased expression of IFN-γ and IL-6 [27].

In bleomycin-induced pulmonary fibrosis in mice, the protective effects of microbial biosurfactants produced by *Bacillus halotolerans* (surfactin) and *Candida parapsilosis* (sophorolipids) showed an impact from antioxidant, anti-inflammatory, and antifibrotic properties via the TGF-β1/Smad-3 pathway [91]. The biosurfactants were able to inhibit the inflammatory and fibrotic responses induced by bleomycin-induced pulmonary fibrosis; however, the effect triggered by surfactin was superior to that of sophorolipids. Nevertheless, neither showed acute toxic effects in the tested animals, nor liver or kidney injuries [91].

These mechanisms converge in consistent evidence of improvement in models of acute and chronic inflammation. In murine models of acute lung injury, surfactin reduced neutrophilic infiltration and tissue damage; in models of chronic inflammation, sophorolipids decreased fibrosis and airway remodeling. These findings reinforce the translational potential of biosurfactants as multifunctional anti-inflammatory agents. Biosurfactants have demonstrated efficacy in models of acute and chronic inflammation by acting simultaneously on physicochemical (reduction of surface tension), immunomodulatory (inhibition of pro-inflammatory pathways), and microbiological (antimicrobial and anti-biofilm action) processes. The combination of these mechanisms generates a multifunctional therapeutic profile, which is reflected in consistent preclinical results.

The evidence discussed throughout this session reinforces that biosurfactants represent a class of biomolecules of great biological and therapeutic relevance, since they act in an integrated way in the modulation of inflammation, in the preservation of alveolar function, and in cellular protection. These compounds broaden the understanding that respiratory diseases, such as asthma, do not exclusively involve airway inflammation, but also profound alterations in alveolar homeostasis and the innate immune response. In this context, biosurfactants emerge as promising candidates for the development of new therapeutic strategies, capable of acting simultaneously on bronchiolar inflammation, alveolar dysfunction, and the cellular mechanisms associated with disease progression, offering a more comprehensive and innovative approach to the treatment of respiratory pathologies.

## Antimicrobial and Antiviral Properties Relevant to Respiratory Diseases

Biosurfactants exhibit a highly relevant antimicrobial and antiviral profile for respiratory applications, combining a broad spectrum of action, the ability to disrupt biofilms, and direct interference with the infectivity of microorganisms. Compounds such as rhamnolipids, surfactin, sophorolipids, and MELs demonstrate significant activity against bacterial, fungal, and viral pathogens that play a central role in the pathophysiology of acute and chronic respiratory diseases.

### Antimicrobial and Antibiofilm Activity

Rhamnolipids, produced by *Pseudomonas* spp., exhibit a strong bactericidal effect against agents of high clinical relevance, such as multidrug-resistant *Pseudomonas aeruginosa*, *Staphylococcus aureus*, and pathogens associated with cystic fibrosis [31]. Their activity derives from their ability to permeabilize and destabilize cell membranes, facilitating bacterial lysis and the dispersion of biofilms that persist in the airways [31]. This property is particularly important in chronic diseases with persistent colonization, where biofilms hinder the effectiveness of conventional antibiotics. Surfactin, a potent lipopeptide from *Bacillus subtilis*, exhibits antimicrobial activity against Gram-positive and Gram-negative bacteria, as well as broad antifungal activity, including efficacy against *Aspergillus fumigatus*, a relevant respiratory pathogen in immunocompromised individuals [92, 93]. Its amphiphilic structure allows direct insertion into microbial membranes, promoting pores and disrupting lipid bilayers.

The significant increase in resistance of pathogenic microorganisms to conventional antimicrobials has driven the search for new antimicrobial agents, directing scientific attention to biosurfactants as potential antibacterial alternatives [94]. Several studies indicate that certain biosurfactants exhibit comparable or superior efficacy to synthetic drugs, combined with a better safety profile, positioning them as promising candidates for therapeutic application in infection control [95].

A lipopeptide biosurfactant produced by the marine microorganism *Bacillus circulans* demonstrated significant antimicrobial activity against *Proteus vulgaris*, *Alcaligenes faecalis*, methicillin-resistant *Staphylococcus aureus* (MRSA), and other multidrug-resistant pathogenic strains, without exhibiting a hemolytic effect, demonstrating a favorable safety profile [96]. Similarly, a rhamnolipid-type biosurfactant, obtained from soybean oil waste, exhibited broad-spectrum antimicrobial activity against various bacteria and fungi, including *Bacillus cereus*, *S. aureus*, *Micrococcus luteus*, *Mucor miehei*, and *Neurospora crassa* [97, 98]. Studies have shown that a lipopeptide antimicrobial substance produced by the *Bacillus subtilis fmbj* strain, composed mainly of surfactin and fengicin, was able to inactivate *Bacillus cereus* endospores. Transmission electron microscopy (TEM) analyses revealed that the lipopeptide promoted structural damage to the spore surface, suggesting a mechanism of action based on the disruption of the integrity of the spore wall and membrane [98].

The potential of surfactin as a feed additive and alternative to antibiotics in the prevention of bacterial diseases in the animal industry was evaluated by Horng *et al. *[99]*.* Surfactin produced by *Bacillus subtilis* and *Bacillus licheniformis* showed antibacterial activity against *Brachyspira hyodysenteriae* and *Clostridium perfringens*, with differences associated with the profile of isoforms produced. Surfactin from *B. subtilis* showed greater efficacy against *B. hyodysenteriae*, while that from *B. licheniformis* exhibited a strong bactericidal effect against *C. perfringens* [99].

Collectively, the combination of antimicrobial and antiviral actions, coupled with the ability to disrupt biofilms and modulate the inflammatory response, positions biosurfactants as highly promising candidates for innovative respiratory therapies. One of the most relevant aspects in the pulmonary context is the ability of biosurfactants to disrupt biofilms, structures that confer great resistance to conventional antimicrobial therapies. In conditions such as cystic fibrosis, biofilms formed by *P. aeruginosa* hinder treatments and aggravate chronic inflammation [100]. Rhamnolipids, produced by *Pseudomonas* spp., are among the most studied compounds and show high efficacy in disrupting the extracellular polymeric substances (EPS). They reduce surface tension and interact with hydrophobic components of the biofilm, promoting bacterial dispersion and increasing permeability to antibiotics, especially aminoglycosides and β-lactams. Studies by Davey *et al.* [100] demonstrated that rhamnolipids significantly decrease the structural cohesion of *P. aeruginosa* biofilms and reduce the density of viable cells. Sophorolipids also exhibit relevant activity against biofilms. Research showed that these glycolipids can prevent the initial adhesion of bacteria to epithelial surfaces, a crucial step in biofilm establishment, as well as disrupt mature biofilms by disrupting the lipid and protein organization of the matrix [101]. Dhadwal and colleagues [101] observed that sophorolipids reduce the thickness and biomass of bacterial biofilms, reinforcing their role as adjuvant agents in respiratory therapies.

### Antiviral Activity and Interference with Viral Infectivity

In addition to antibacterial and antifungal properties, several biosurfactants exhibit antiviral activity, especially against enveloped viruses. Surfactin is one of the most studied molecules in this context, having demonstrated the ability to inhibit viral entry by destabilizing the lipid envelope of viruses such as influenza, respiratory syncytial virus, and coronavirus [102–105]. The mechanism involves strong surfactant action, which disrupts the viral lipid bilayer and prevents fusion with the host cell membrane, suggesting therapeutic potential not only in acute infections but also as a prophylactic agent in inhaled formulations.

The antiviral activity of biosurfactants, especially surfactin and its analogues, has been extensively described in the literature. These compounds demonstrate greater efficacy against enveloped viruses, such as retroviruses and herpesviruses, compared to non-enveloped viruses, suggesting that the main mechanism of action involves physicochemical interactions with the viral envelope, leading to its destabilization [103]. Surfactin produced by *Bacillus subtilis* has demonstrated potent antiviral activity against enveloped viruses by acting as a membrane fusion inhibitor. At relatively low concentrations (15–50 μg/mL), surfactin suppressed the proliferation of two swine viruses (porcine epidemic diarrhea virus and transmissible gastroenteritis virus) in epithelial cells without exhibiting cytotoxicity or causing viral envelope rupture, indicating that surfactin significantly reduces virus fusion to the cell membrane through its insertion into the lipids of the viral envelope, preventing the formation of the negative curvature necessary for fusion [102]. Lipopeptides produced by *Bacillus subtilis* fmbj were able to inactivate different animal viruses and inhibit viral replication in some cases, although this activity varies according to the type of virus [106]. Furthermore, sophorolipids have shown activity against the human immunodeficiency virus (HIV) [107], and rhamnolipids, isolated from *Pseudomonas* sp., have shown a significant antiviral effect against herpes simplex viruses types 1 and 2, in a dose-dependent manner and at concentrations below the critical micelle concentration [108].

The antiviral potential of surfactin against severe acute respiratory syndrome coronavirus 2 (SARS-CoV-2) was tested *in vitro* in a cell model of infection. Treatment with surfactin for 2 h was able to reduce the infectivity of SARS-CoV-2 in Vero E6 cells, both at 24 h and after 7 days of viral inoculation, probably compromising the integrity of the viral membrane, on the other hand no cytotoxic activity of surfactin was observed [105].

**Fig. 4:**
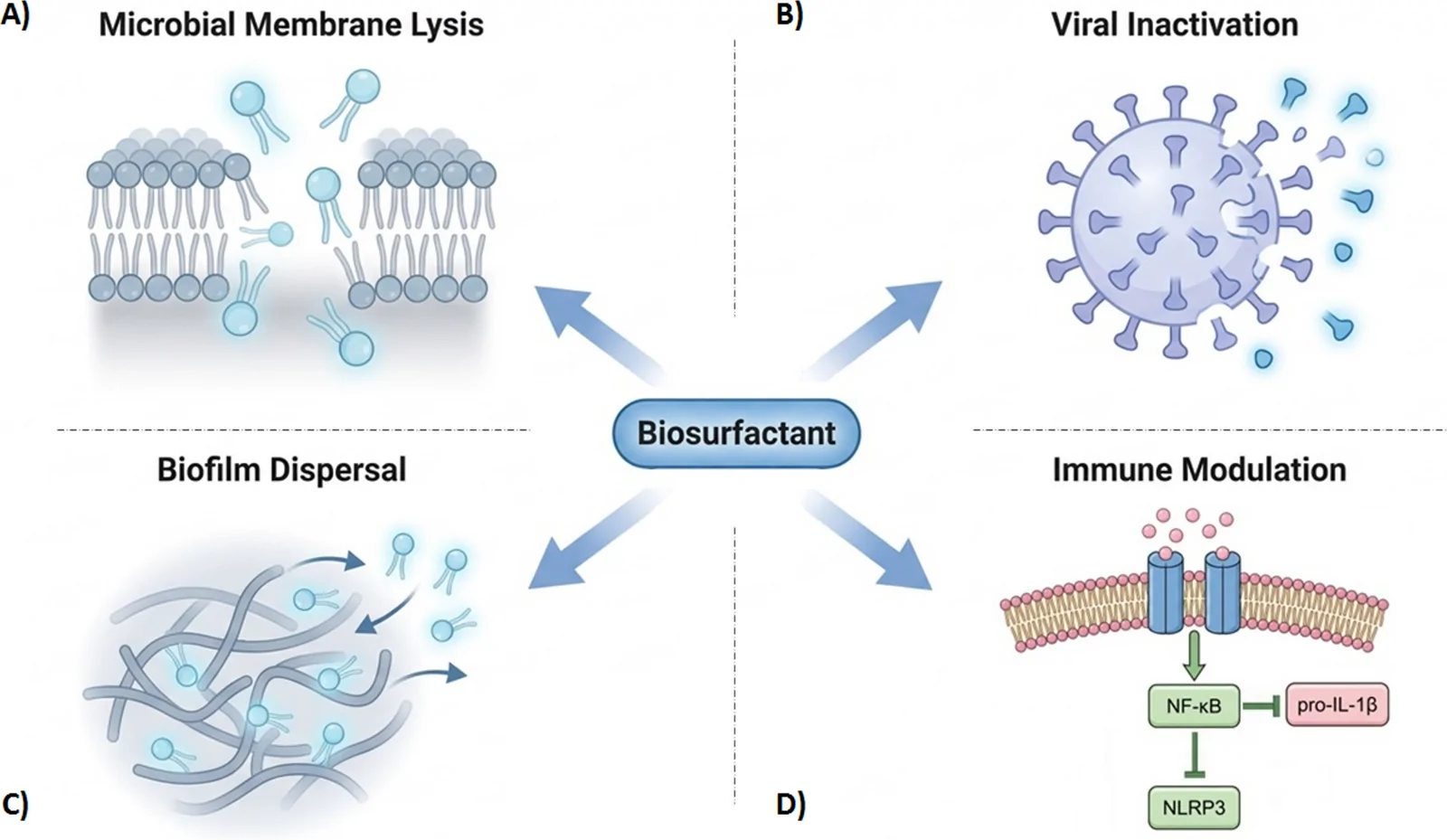
Main mechanisms of action of biosurfactants: (**A**) Lysis of the microbial membrane demonstrating its antimicrobial action. (**B**) Inactivation of the viral envelope related to its antiviral action. (**C**) Biofilm dispersion demonstrating its antibiofilm action. (**D**) Immunomodulation showing its potential in attenuating the inflammatory response.

Taken together, the evidence indicates that biosurfactants constitute a multifunctional platform of interest for controlling respiratory infections, combining antibacterial and antifungal activity, biofilm disruption, and interference with the infectivity of certain viruses, particularly enveloped ones. This combination of properties may be particularly relevant in conditions characterized by the coexistence of persistent microbial colonization, biofilm formation, and chronic inflammation. Furthermore, emerging evidence elucidates the relationships between molecular structure, concentration, mechanism of action, selectivity, and safety for respiratory applications, reinforcing their therapeutic potential (Fig. 4).

## Other Biotechnological Applications of Biosurfactants

### Biomedical Applications Beyond Respiratory Diseases

The structural and amphipathic properties of biosurfactants contribute significantly to their biological activities, including anticancer activity. The biological effects of biosurfactants have been extensively investigated in different cell models, including tumor cell lines, with evidence of cytotoxic, antiproliferative, and modulatory activities on cellular processes such as apoptosis and tumor progression [39].

**Fig. 5:**
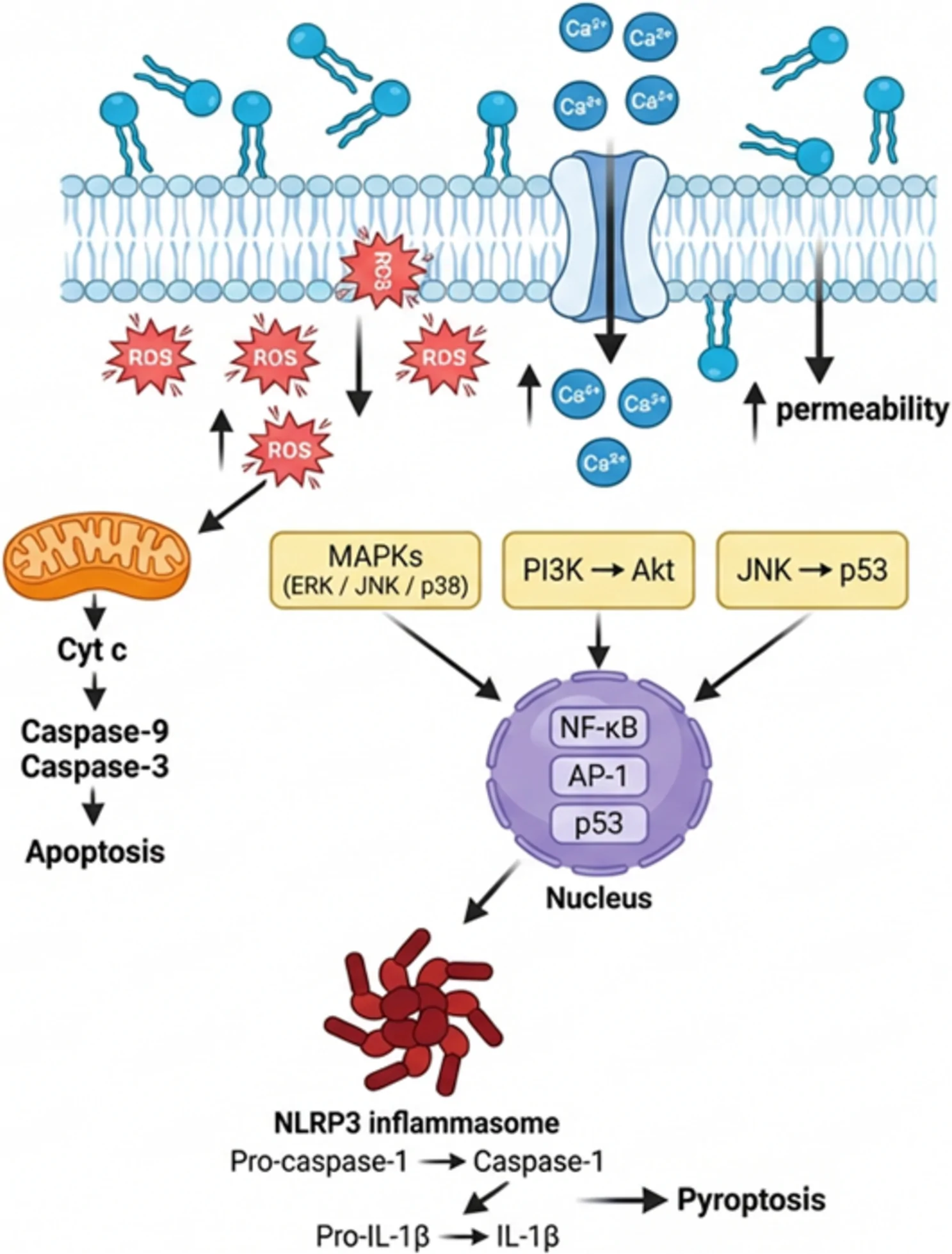
Cell signaling pathways triggered by the interaction of biosurfactants with the plasma membrane. Biosurfactant molecules interact with the lipid bilayer of the plasma membrane, leading to increased membrane permeability, calcium (Ca^2^⁺) influx, and increased production of reactive oxygen species (ROS). This activates intracellular signaling cascades, including the MAPK pathways (ERK, JNK, and p38), the PI3K/Akt pathway, and the JNK-p53 axis, which trigger nuclear translocation and activation of transcription factors such as NF-κB, AP-1, and p53, resulting in the regulation of gene expression related to inflammation. This contributes to the activation of the NLRP3 inflammatory complex, leading to caspase-1 activation, maturation of pro-IL-1β into IL-1β, and induction of pyroptosis. In parallel, ROS leads to mitochondrial dysfunction, characterized by the release of cytochrome c (Cyt c), and consequently the activation of caspase-9 and caspase-3, promoting cell death by apoptosis.

The proposed mechanisms for the action of biosurfactants in anticancer activity *in vitro* include inhibition of cell growth [109], cell cycle arrest [109], induction of programmed cell death (apoptosis) [110], and modulation of processes associated with tumor progression, such as invasion, migration, and colony formation [111]. Evidence indicates that treatment with some biosurfactants, mainly surfactin, compromises the viability of cancer cells by inactivating signaling pathways related to cell survival and regulating key cell cycle proteins, resulting in the blockage of proliferation [112]. Furthermore, surfactin can induce apoptosis through the intrinsic mitochondrial pathway, associated with increased production of reactive oxygen species (ROS), alterations in mitochondrial membrane potential, and an imbalance in the Bax/Bcl-2 ratio, culminating in the release of cytochrome c and activation of caspases [112]. Additionally, surfactin exhibits anti-invasive and anti-metastatic effects, associated with the modulation of matrix metalloproteinase expression, such as MMP-9, and the inhibition of pro-tumor signaling pathways, including NF-κB, AP-1, PI3K/Akt, and ERK1/2 [111] (Fig. 5).

Cytotoxic activities are very evident in breast cancer, where surfactin produced by the *B. subtilis* (573) strain promoted a significant reduction in cell viability in T47D and MDA-MB-231 cell lines in a dose- and time-dependent manner, reaching approximately 50% inhibition at 193 μM after 48 and 72 h, respectively. Additionally, the inhibition of cell proliferation was accompanied by an increase of approximately 10% in cell cycle arrest in the G0/G1 phase after treatment with surfactin at this concentration [109]. In another study, Cao and colleagues [110] reported dose- and time-dependent inhibition of proliferation of human breast cancer cells MCF-7, with IC₅₀ values of 82.6 μM at 24 h, 27.3 μM at 48 h, and 14.8 μM at 72 h. And induced cell death was characterized as apoptotic, based on typical morphological changes identified by acridine orange/ethidium bromide staining and the TUNEL assay [110]. Surfactin treatment also promoted an increase in intracellular Ca^2^⁺ concentration. Likewise, flow cytometry revealed time-dependent apoptosis, accompanied by cell cycle arrest in the G2/M phase. Meanwhile, western blot analysis demonstrated an accumulation of p53 and p21, associated with inhibition of the cyclin B1/p34 complex. Taken together, the surfactin suppressed MCF-7 cell proliferation by inducing apoptosis and cell cycle arrest, possibly mediated by increased intracellular Ca^2^⁺ and modulation of key cell cycle regulators [110].

Surfactin was also able to inhibit proliferation and induce apoptosis in MCF-7 human breast cancer cells through molecular mechanisms dependent on the generation of reactive oxygen species (ROS). Surfactin treatment promoted increased ROS and sustained activation of the ERK1/2 and JNK signaling pathways, but not the p38 pathway, with cell death being reversed by antioxidants and specific ERK1/2 and JNK inhibitors. JNK activation was shown to be ROS-dependent and essential for the observed apoptotic effects. Furthermore, surfactin triggers the mitochondrial/caspase apoptotic pathway, characterized by an increased Bax/Bcl-2 ratio, loss of mitochondrial membrane potential, cytochrome c release, and caspase activation, events that are blocked by antioxidants, JNK inhibitors, and a general caspase inhibitor, indicating that surfactin-induced apoptosis occurs predominantly via a mitochondrial/caspase pathway mediated by the ROS/JNK axis [112].

Another effect showed that surfactin was able to exhibit antimetastatic effects *in vitro* by inhibiting the invasion, migration, and colony formation of human breast carcinoma cells. These effects were associated with suppression of MMP-9 expression and activity, as well as inhibition of the activation and nuclear translocation of the transcription factors NF-κB and AP-1. Additionally, surfactin repressed the phosphorylation of the PI3K/Akt and ERK pathways, whose inhibition also resulted in reduced MMP-9 expression. The role that surfactin plays in antimetastatic activity through the modulation of multiple pro-tumor signaling pathways is evident, highlighting its potential as a therapeutic agent in the context of breast cancer metastasis [111].

Similar cytotoxic effects have been described in different types of cancer, including inhibition of cell growth and cell cycle arrest [109], induction of programmed cell death by apoptosis [110], as well as modulation of processes associated with tumor progression, such as invasion, migration, and colony formation, reinforcing the broad and multifaceted nature of surfactin's antitumor action. Cytotoxic effects were also determined by Abdelli *et al.* [113] in breast cancer cells (T47D) and murine melanoma cells B16F10, with IC50 values of 0.66 mg/mL and 1.17 mg/mL, respectively.

Sophorolipids have also shown potential to induce different forms of cell death in various types of cancer. In HepG2 cell culture, sophorolipids were able to inhibit cell growth and induce programmed cell death (apoptosis) through activation of the caspase-3 pathway [114]. The same biosurfactant, sophorolipid, in human liver cancer cells H7402 was able to induce apoptosis by blocking the cell cycle in the G1 and, partially, S phases, associated with caspase-3 activation and increased cytoplasmic Ca^2^⁺ concentration [115]. Biological assays in MDA-MB-231 breast cancer cells with C18:0 and C18:1 lactonic sophorolipids (SL) showed high cytotoxicity, while C18:3 SL exhibited an intermediate cytotoxic effect, but superior to the acidic SLs. In this context, the lactone SL C18:1 was able to inhibit cell migration without compromising viability, in addition to increasing the intracellular production of reactive oxygen species, highlighting its potential as an antitumor agent [116].

Sophorolipids derived from non-pathogenic yeasts, such as *Starmerella bombicola*, have demonstrated significant anticancer potential, going beyond traditional *in vitro* 2D monolayer breast cancer models. When evaluated in more complex three-dimensional models, such as 3D spheroids, the IC₅₀ values indicated that one of the sophorolipids exhibited toxicity similar to that of doxorubicin, used as a chemotherapeutic control [117].

Regarding biosurfactants that may have more than one sugar in their composition, such as rhamnolipids (RL), interestingly, the anticancer activity seems to depend on the structural differentiation between mono-RLs and di-RLs, suggesting that the hydrophilic portions of RLs are primarily responsible for the anticancer chemotherapeutic activity [118]. Breast cell lines (MCF-10A, MCF-7, and MDA-MB-231) were treated with RLs in combination with cisplatin, and unlike healthy cells, both cancer cell lines tested showed sensitivity to treatment with RLs [118]. For rhamnolipids produced by *Pseudomonas aeruginosa* MR01, fractionated into mono- and di-rhamnolipids and evaluated for cell viability, a dose-dependent decline was observed, with IC₅₀ values of 25.87 μg/mL and 31.00 μg/mL for mono- and di-rhamnolipids, respectively, after 48 h of treatment in MCF-7 cells [119]. Characteristic signs of apoptosis were identified in this cell model, both by the morphology observed in reversed-phase contrast microscopy and by the fluorescence after Hoechst staining. Taken together, the rhamnolipids derived from the MR01 strain showed relevant anticancer potential against MCF-7 cells, justifying further investigations for their development as natural antitumor agents. Apparently, the mono-rhamnolipid structure seems to favor more efficient cellular interactions, resulting in greater cytotoxicity and highlighting a relevant structure–function relationship for this type of bioactivity [119]. The cytotoxicity of rhamnolipids produced by *Pseudomonas* was also evaluated in the MDA-MB-231 breast cancer cell line, resulting in an IC₅₀ of 0.05 μg/mL after 72 h of treatment, an extremely low value and particularly relevant for a triple-negative breast tumor, known for its more aggressive behavior [120]. In different cell lines, such as colorectal cancer cell cultures (HCT-116 and Caco2), the mono-rhamnolipid biosurfactant also reduced the viability of both cell lines. After 24 h of treatment with mono-RL, the viability of HCT-116 and Caco2 cell lines was reduced to a range of 14.99 to 12.16% of untreated cells at concentrations between 10 and 100 µg mL⁻^1^ [121]. This showed the importance of evaluating different cell lines and distinct structural forms of biosurfactants, since the biological and molecular variability between models and molecules can reveal essential details about their activity and guide the development of more effective therapeutic agents. Another activity observed for rhamnolipid biosurfactants was their ability to increase cell permeability. Evaluated in Caco2 culture, rhamnolipid was able to modulate cell integrity and increase paracellular permeability in cells within monolayers [122]. Rhamnolipids can represent a new class of molecules with potential use as permeabilization enhancers, employed to increase the bioavailability of therapeutic drugs.

Other biosurfactants also exhibit cytotoxic properties, exemplified by the glycolipid mannosylerythritol-B, capable of triggering apoptosis in murine melanoma B16F10 cells at concentrations from 20 µg/mL, an effect directly associated with increased intracellular production of reactive oxygen species [123]. Meanwhile, the biosurfactant MELs, produced from vegetable oil by *Pseudozyma aphidis*, promoted growth inhibition and apoptosis induction in B16 melanoma cells in a dose-dependent manner, in addition to triggering cell cycle arrest in the S phase. Further analyses by quantitative RT-PCR and western blotting showed a gradual increase in the mRNA and protein expression of Caspase-12, CHOP, GRP78, and Caspase-3, accompanied by the downregulation of Bcl-2. Considering also the activation of IRE1 and ATF6 signaling pathways, the data would suggest that MEL-A induced apoptosis in B16 cells would be associated with the activation of the endoplasmic reticulum stress response [124]. Rationally developed MELs analogs have demonstrated selective cytotoxicity against human skin cancer cells, with particular emphasis on high selectivity against HSC-5 squamous cell carcinoma cells. The observed results indicated that R-MTL-A, a synthetic analogue of MEL-A, induced cell death with necrosis-like characteristics in these cells, possibly through the reduction of plasma membrane fluidity. Additionally, R-MTL-A showed an efficient reparative effect on damaged skin cells, suggesting its potential as a lead compound for the development of new cosmeceuticals with dual action: selective cytotoxicity for tumor cells and regenerative properties for injured skin cells [125].

Consistently, similar cytotoxic effects have been described in different types of cancer, including inhibition of cell growth and cell cycle arrest, induction of programmed cell death by apoptosis, as well as modulation of processes associated with tumor progression, such as invasion, migration, and colony formation, reinforcing the broad scope of the antitumor action of biosurfactants.

One study evaluated different types of microbial glycolipids, sophorolipids (SLs, in acidic and lactonic forms) and rhamnolipids (RLs, mono-RL and di-RL), in addition to a synthetic surfactant (SLES), using the HaCaT human keratinocyte cell line stimulated with LPS as an experimental model to investigate safety and bioactivity in dermocosmetic applications [126]. The results demonstrated that the acidic SL and mono-RL showed minimal effects on cell viability, morphology, and the production of pro-inflammatory cytokines, indicating a good safety profile. At non-cytotoxic concentrations, di-RL exerted an immunomodulatory effect, significantly reducing IL-8/CXCL8 production and increasing IL-1RA/IL-1RN expression. In an integrated manner, the results indicated microbial glycolipids as safe, functionally active, and promising alternatives compared to synthetic surfactants, with applicability in both dermocosmetics and in modulating the immune response in inflammatory skin diseases such as psoriasis [126].

Biosurfactants can modulate the microbiota, decrease pathogen colonization, and reduce inflammation, leading to improved zootechnical performance and greater food safety. The strategic role of biosurfactants in reducing antibiotic use and addressing antimicrobial resistance, positioning them as promising tools for more sustainable animal production systems that comply with current regulatory requirements [127]. Tsai and colleagues highlight inflammatory bowel disease (IBD), a non-infectious condition characterized by chronic inflammation of the gastrointestinal tract that remains a relevant clinical challenge. The study evaluated the therapeutic potential of fermented *Bacillus licheniformis* products containing surfactin (SBLF) and commercial surfactin (CS) in the treatment of dextran sodium sulfate (DSS)-induced colitis in a murine model. The results demonstrated that SBLF was able to improve the IBD condition, reducing chronic inflammation and inhibiting the activation of the NLRP3 inflammasome, suggesting its potential as a nutraceutical for human use or as a feed additive for production and companion animals in the prevention of IBD [127]. New insights are constantly emerging with a highly dynamic and versatile molecule like the lipopeptide surfactin; the work of Jia *et al.* [128] showed that surfactin from *Bacillus subtilis* improves the immune response and contributes to the maintenance of intestinal microbiota homeostasis, indicating superior nutrient absorption in a murine model.

### Biotechnological Platforms and Delivery Systems

An important advance in the healthcare field was the development of a self-assembled nanoparticle system based on rhamnolipids, loaded with pheophorbidin α (Pba), a hydrophobic photosensitizer. These nanoparticles exhibited rapid internalization into SCC7 tumor cells and induced photodynamic damage after laser irradiation. In a murine model with SCC7 tumor, intravenous administration resulted in prolonged circulation time and high tumor accumulation, as demonstrated by real-time fluorescence imaging [129]. After irradiation, the rhamnolipid nanoparticles promoted complete tumor suppression by *in vivo* photodynamic therapy. The great potential of rhamnolipid-based nanoparticles as drug delivery platforms indicates that further investments in research on these molecules could lead to significant advances [129].

In biofilm models, co-administration of rhamnolipids with antibiotics resulted in synergistic effects with increased drug penetration and reduced bacterial viability. The biosurfactant can increase the efficacy of different antibiotics by altering the permeability of the bacterial membrane and facilitating drug entry into cells. This combination of rhamnolipids with antibiotics may represent a promising strategy to enhance antimicrobial therapies and reduce bacterial resistance, which could be expanded to other combinations [130].

Safety and toxicological description are crucial for clinical translation. In general, biosurfactants such as MELs and sophorolipids exhibit low *in vitro* cytotoxicity and good biodegradability in preclinical studies, while surfactin and some rhamnolipids may exhibit greater hemolytic activity at high concentrations, requiring careful formulation and dosage [38, 59]. Toxicological assessments should include cytotoxicity tests in respiratory epithelial cells, evaluation of acute and chronic inflammation in animal models, genotoxicity, immunogenicity, and potential for inducing bronchospasm or hypersensitivity. Purification procedures that remove microbial endotoxins and metabolic byproducts are fundamental to ensuring safety. The therapeutic potential of biosurfactants is high, but translation requires rigorous technical development, standardization, comprehensive toxicological studies, and clinical trials that prove efficacy and safety in target populations.

The amphipathic characteristics of surfactin position it as a promising option for applications in nanotechnology, especially in the areas of drug delivery, antimicrobial formulations, and food preservation. Thus, nanoformulations represent a promising strategy to improve the anticancer effect of surfactin, as well as other biosurfactants in general [39]. In general, therapeutic and technological applications of biosurfactants in the pulmonary context involve approaches ranging from inhalable formulations to their use as a multifunctional excipient in combination therapies. The possibility of incorporating biosurfactants into inhalable formulations and nanoparticulate systems is attractive because these molecules can reduce surface tension, promote particle dispersion, and improve interaction with the alveolar epithelium, increasing local drug delivery and reducing adverse systemic effects. Studies demonstrated that nanoparticles coated with surfactin or rhamnolipids improved the stability of structures and increased the controlled release of active agents, in addition to conferring intrinsic antimicrobial activity to the delivery system [39, 129]. However, technical challenges include standardization of composition, physicochemical stability in nebulizers, and optimization of pulmonary deposition.

## Challenges and Future Perspectives

Despite promising preclinical evidence, several challenges must be overcome before biosurfactants can be developed into clinically viable respiratory therapies. Although various classes exhibit promising biological properties, the transition from laboratory scale to pilot and industrial processes remains constrained by low yields and productivity, high substrate costs, excessive foaming, oxygen and mass transfer difficulties, fermentation control issues, and the complexity of recovery and purification steps. These factors are particularly relevant for surfactin and rhamnolipids, as their production in bioreactors can face limitations related to foam formation and the need for strictly controlled operating conditions [131–135].

Structural standardization poses a particularly significant challenge for pulmonary pharmaceutical applications. Biosurfactants such as surfactin, rhamnolipids, and sophorolipids can be produced as mixtures of isoforms and congeners with varying lipid chain lengths and degrees of saturation, characteristics that can substantially alter their surfactant, antimicrobial, and anti-inflammatory activities, as well as their toxicological profiles. Consequently, scale-up efforts must address not only increasing production titers but also ensuring consistency in molecular composition and biological activity across different batches. For therapeutic applications, it is essential to establish quality parameters covering molecular identity, purity, isoform distribution, biological activity, the absence of endotoxins and microbial contaminants, and stability during storage and administration [136, 137].

In this context, sophorolipids are currently at a relatively advanced stage of industrial development, with processes already established at a commercial scale and recent investments aimed at expanding production capacity. However, experience with sophorolipids does not eliminate the challenges faced by other classes, as productivity, cost, product composition, substrate availability, and regulatory requirements remain decisive factors for their use in higher value-added applications, including the pharmaceutical sector [134, 138].

For surfactin and rhamnolipids, the development of economically competitive processes remains particularly relevant. In the case of rhamnolipids, costs associated with fermentation and purification still represent significant obstacles to competitiveness against synthetic surfactants. Strategies based on strain engineering, the use of low-cost renewable raw materials, metabolic optimization, process intensification, and integrated product recovery can contribute to reducing these costs [135, 139].

There is pharmaceutical market space for new pulmonary surfactants, especially in niche respiratory diseases that still lack effective or optimized treatments. Although exogenous surfactants are widely used in the treatment of neonatal respiratory distress syndrome (RDS), there are other respiratory conditions where surfactant modulation or replacement may be promising. For example, exogenous surfactants have been studied for respiratory diseases, especially in adults with conditions such as ARDS, a serious condition often associated with severe infections or lung trauma. Current formulations are not ideal, and new formulations may perform better in these scenarios, with improved surfactant efficacy [140, 141]. In other conditions such as asthma and COPD, although research on the role of surfactants in these diseases is still in the early stages of development, surfactant modulation may be useful in improving lung function and controlling inflammation, especially in severe cases or those resistant to standard treatments [142]. In pulmonary infections or the COVID-19 pandemic, there has been renewed interest in therapies that could improve lung function in severe cases of viral infection. In this context, the development of new pulmonary surfactants with anti-inflammatory or immunomodulatory effects would have a significant impact on the treatment of respiratory complications caused by severe infections [143]. In terms of technological advancements, it is believed that there is great potential for the development of surfactants with advanced properties, such as greater stability, prolonged half-life in the lungs, and better distribution throughout the airways. These improvements could meet a clinical demand for more effective treatments, especially those targeting adult patients [144]. In nanotechnology and combination therapies, new surfactants can be developed using nanotechnology to improve administration and efficacy, or combined with other therapies, such as anti-inflammatories or antimicrobials, to treat complex diseases like cystic fibrosis or ARDS [145]. Therefore, new pulmonary surfactants that meet new clinical demands may have a promising place in the market, especially if they are targeted at adult diseases and emerging respiratory pathologies.

In the clinical setting, there is still a scarcity of robust clinical trials, with most evidence limited to *in vitro* or preclinical studies. Translation to humans requires assessments of pulmonary toxicity, biodistribution, impact on the respiratory microbiota, immunogenicity, and efficacy against inflammation and actual infections. Systematic trials are essential to confirm safety and efficacy, as well as to guide dosage parameters and routes of administration. There is also a need for predictive models capable of evaluating the interaction of biosurfactants with human lung tissue, including organoid models, lung-on-a-chip systems, and lung tissue explants. These models would provide greater accuracy in simulating physiological and pathological conditions, enabling the prediction of clinical effects with greater reliability.

Finally, prospects include the potential for personalized therapies and applications in regenerative medicine. Due to their immunomodulatory, antimicrobial, and bioactive properties, biosurfactants can be integrated into biomaterials, scaffolds, and controlled-release systems for lung tissue regeneration or for therapies tailored to individual inflammatory and infectious profiles. Advances in biotechnology, nanotechnology, and pharmacology are expected to substantially expand the scope of these molecules as next-generation therapeutic tools.

## Conclusion

The evidence discussed in this review indicates that biosurfactants constitute a promising class of biomolecules for the development of new therapeutic strategies for respiratory diseases, owing to their ability to integrate various biologically important properties, including surfactant activity, antimicrobial and antibiofilm action, antiviral activity, modulation of the inflammatory response, and, in some cases, antioxidant effects. This multifunctionality is particularly relevant in the pulmonary context, where alterations in surface tension, persistent inflammation, oxidative stress, recurrent infections, and biofilm formation often coexist and contribute to the progression and severity of respiratory diseases.

Among the compounds evaluated, surfactin exhibits the most comprehensive preclinical profile, encompassing evidence regarding surface tension reduction, alveolar stabilization, improved respiratory mechanics, antimicrobial and antiviral activity, and the modulation of inflammatory pathways, including TLR4, NF-κB, MAPK, Akt, and inflammasomes. Rhamnolipids, in turn, are notable primarily for their potent antimicrobial and antibiofilm activity, which is particularly relevant for persistent respiratory infections associated with *Pseudomonas aeruginosa* colonization. Meanwhile, sophorolipids demonstrate a promising profile of antimicrobial, antibiofilm, and immunomodulatory activity, whereas MELs stand out for their biocompatibility, biodegradability, and anti-inflammatory and antioxidant potential. However, for these latter classes, evidence specifically related to pulmonary physiology remains more limited, underscoring the need for targeted studies. A particularly relevant aspect is that the therapeutic potential of biosurfactants should not be interpreted solely in terms of replacing endogenous pulmonary surfactant. Although the ability to modulate air–liquid interfaces and reduce surface tension can contribute to alveolar stability and respiratory mechanics, their antimicrobial, antibiofilm, and immunomodulatory effects significantly expand their potential applications. Thus, these molecules may represent a multifunctional platform capable of simultaneously acting on different components of pulmonary pathophysiology, especially in conditions where inflammation, infection, and respiratory barrier dysfunction are interrelated.

Despite promising preclinical evidence, clinical translation remains limited by the lack of systematic studies on safety, efficacy and formulation. Standardization and molecular characterization also remain essential requirements to ensure reproducibility, safety, and therapeutic performance. Therefore, future studies should prioritize experimental models that are more representative of human pulmonary physiology. Thus, although still far from definitive clinical application, biosurfactants represent an innovative frontier with high translational potential to improve lung function and control inflammation, justifying further multidisciplinary research in this area.
